# Development of 3D-Printable Lead-Free Composite Materials for Mixed Photon and Neutron Attenuation

**DOI:** 10.3390/polym18020176

**Published:** 2026-01-08

**Authors:** Shirin Arslonova, Jurgita Laurikaitiene, Diana Adliene

**Affiliations:** Department of Physics, Kaunas University of Technology, Studentu Str. 50, LT51368 Kaunas, Lithuania; jurgita.laurikaitiene@ktu.lt

**Keywords:** 3D-printed polymer composites, mixed photon–neutron shielding, lead-free composites

## Abstract

The growing use of radiation technologies has increased the need for shielding materials that are lightweight, safe, and adaptable to complex geometries. While lead remains highly effective, its toxicity and weight limit its suitability, driving interest in alternative materials. The process of 3D printing enables the rapid fabrication of customized shielding geometries; however, only limited research has focused on 3D-printed polymer composites formulated specifically for mixed photon–neutron fields. In this study, we developed a series of 3D-printable ABS-based composites incorporating tungsten (W), bismuth oxide (Bi_2_O_3_), gadolinium oxide (Gd_2_O_3_), and boron nitride (BN). Composite filaments were produced using a controlled extrusion process, and all materials were 3D printed under identical conditions to enable consistent comparison across formulations. Photon attenuation at 120 kVp and neutron attenuation using a broad-spectrum Pu–Be source (activity 4.5 × 10^7^ n/s), providing a mixed neutron field with a central flux of ~7 × 10^4^ n·cm^−2^·s^−1^ (predominantly thermal with epithermal and fast components), were evaluated for both individual composite samples and layered (sandwich) configurations. Among single-material prints, the 30 wt% Bi_2_O_3_ composite achieved a mass attenuation coefficient of 2.30 cm^2^/g, approximately 68% of that of lead. Layered structures combining high-Z and neutron-absorbing fillers further improved performance, achieving up to ~95% attenuation of diagnostic X-rays and ~40% attenuation of neutrons. The developed materials provided a promising balance between 3D-printability and dual-field shielding effectiveness, highlighting their potential as lightweight, lead-free shielding components for diverse applications.

## 1. Introduction

Nuclear technology represents one of the most significant scientific achievements of the modern era, providing immense benefits across diverse sectors, including medical diagnostics, nuclear power generation, radiation processing, and advanced imaging technology [1,2,3,4]. However, the proliferation of these applications results in the inevitable production of harmful ionizing radiation, such as X-rays, gamma rays (photons), and neutrons [5,6,7,8]. Exposure to these high-energy radiations poses serious hazards to human health, potentially causing irreversible biological damage, including cancer induction, genetic mutations, brain malfunction, and digestive and other systemic complications [9,10,11,12,13]. Consequently, ensuring effective protection from radiation is essential to minimize occupational exposure and reduce environmental risks. The preferred method for achieving this safety objective is the implementation of effective shielding materials, mitigating exposure more reliably than relying solely on the limitations of time or distance from the source [14,15,16,17].

Historically, heavy metals like lead have served as the principal materials for photon shielding due to their high density and atomic number (Z), offering robust attenuation capacity against gamma rays and X-rays [18,19,20]. However, conventional lead-based shields pose several critical disadvantages, prompting an urgent need for alternatives. Lead is a heavy, toxic material linked to environmental contamination and severe health consequences, including neurological damage. Moreover, it causes recycling problems and is notably ineffective for shielding neutrons [21,22,23,24,25,26].

This demand for safer, non-toxic, lightweight, and high-efficiency radiation protection solutions has driven the investigation of polymer matrix composites (PMCs) as compelling alternatives [27,28]. Polymers offer numerous advantages over metallic or concrete shields, including excellent processing performance, resistance to corrosion, inherent flexibility, thermal stability, and low density, which is advantageous for mobile and aerospace applications. While polymers alone typically possess insufficient attenuation capacity for high-energy photons due to their low-Z composition, their performance can be radically transformed by incorporating suitable heavy metal fillers [29,30,31,32,33].

For photon (X-ray and gamma-ray) shielding, the focus has shifted toward high-atomic number (high-Z) elements such as Tungsten (W) and Bismuth (Bi) and their compounds, which are favored for their high density and effectiveness in promoting photoelectric absorption at low energies [29,32,34,35,36]. Various studies have demonstrated the viability of integrating these high-Z fillers into polymer matrices like epoxy resin, HDPE, or even highly temperature-resistant polyether ether ketone (PEEK) to achieve superior gamma attenuation comparable to or exceeding lead, without compromising mechanical stability [37,38,39].

Conversely, effective neutron shielding fundamentally relies on two distinct processes: fast neutron moderation and thermal neutron absorption. Hydrogen-rich polymers, particularly polyethylene (PE), serve as indispensable matrices for moderating fast neutrons via elastic scattering due to their high hydrogen concentration. Once slowed, thermal neutrons must be captured by isotopes possessing huge neutron absorption cross-sections [40,41]. Boron compounds, such as boron carbide (B_4_C) and boron nitride (BN), are traditionally employed, yielding low-energy secondary gamma rays [42,43]. However, gadolinium oxide (Gd_2_O_3_) is increasingly utilized, given that gadolinium (Gd) boasts significantly higher thermal neutron absorption cross-sections [44,45]. These materials form composites, such as Gd_2_O_3_/PE and BN/HDPE nanocomposites, which show enhanced thermal neutron absorption and mechanical properties [44,46].

The development of materials capable of robust mixed neutron–photon shielding is particularly complex, necessitating a careful combination of light elements (for moderation and absorption) and heavy elements (for photon and secondary gamma attenuation). A critical challenge persists in achieving the homogeneous dispersion of fillers with widely differing densities, often leading to phase segregation and reduced overall composite performance, especially when manufacturing materials for simultaneous dual-field protection. This complexity often mandates intricate layering or the design of hybrid core–shell nanoparticles to maximize efficacy [46,47,48,49].

The manufacturing technology itself has also become a focal point of innovation. Additive manufacturing (AM), commonly known as 3D printing, particularly fused deposition modeling (FDM), offers exceptional advantages for creating customized shields with complicated geometries that maximize material efficiency and minimize waste. FDM allows for rapid prototyping and the ability to control filler distribution or orientation layer-by-layer, addressing some limitations faced by traditional molding techniques. Consequently, many contemporary research efforts involve integrating high-Z elements (W, Bi) and neutron absorbers (B, Gd) within printable polymer matrices, including acrylonitrile butadiene styrene (ABS) and PEEK, to produce custom, functional shielding accessories [50,51,52,53,54]. However, a significant gap remains in the comprehensive development of 3D-printable, lightweight, lead-free composites that reliably deliver high-efficiency performance across both neutron and high-energy photon spectra simultaneously [55,56,57,58,59].

Therefore, the main aim of this work was to develop and thoroughly characterize 3D-printable, lead-free, lightweight composite materials using an acrylonitrile butadiene styrene (ABS) matrix reinforced with a combination of boron nitride (BN), gadolinium oxide (Gd_2_O_3_), bismuth oxide (Bi_2_O_3_), and tungsten (W) fillers, specifically optimized for mixed neutron and photon radiation shielding applications.

## 2. Materials and Methods

### 2.1. Materials and Composite Formulations

Acrylonitrile–butadiene–styrene (MAGNUM™ 3453 ABS) ((C_8_H_8_·C_4_H_6_·C_3_H_3_N)_n_), selected as the matrix material due to its good processability and compatibility with extrusion-based 3D printing, was purchased from 3Devo (3Devo, Utrecht, The Netherlands). Functional fillers, including Bi_2_O_3_ (99.99%, ~300 nm), Gd_2_O_3_ (99.5%, <200 nm), W powder (99.9%, ≤300 nm), and BN (98%, <500 nm), were obtained from VI Halbleiter material GmbH (Laatzen, Germany). The key physical parameters of the raw materials and the corresponding composite formulations are summarized in Table 1.

Five material compositions were investigated: pure ABS as the reference material and four ABS-based composites containing BN, W, Gd_2_O_3_, and Bi_2_O_3_ at the respective loadings listed in Table 1. The selected filler contents were mainly chosen based on filament extrusion stability and FDM printability. Increasing the filler loading resulted in higher melt viscosity, brittle filament behavior, and unstable extrusion, as commonly reported for highly filled polymer composites in the literature review. Therefore, filler concentrations were limited to levels that allowed continuous filament production and reliable printing under identical conditions. Pure ABS was printed to provide a baseline for evaluating the influence of filler incorporation on printability, density, and radiation attenuation. Fillers represent both high-Z photon attenuators (Bi_2_O_3_, W, Gd_2_O_3_) and neutron-reactive materials (BN, Gd_2_O_3_), enabling a comparative assessment of their individual contributions to the shielding performance of the 3D-printed composites.

### 2.2. Preparation of Composite Filaments

Composite filaments were produced by dry-mixing ABS granules with the corresponding fillers for 15–20 min, followed by extrusion using a 3Devo Precision (3Devo, Utrecht, The Netherlands). The extruder was purged with DevoClean HDPE before each formulation to prevent cross-contamination.

**Figure 1:**
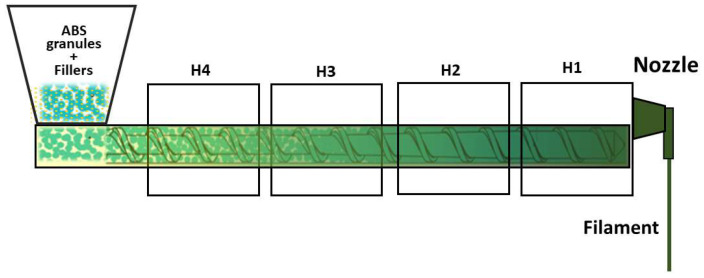
Schematic overview of the extrusion setup used for composite filament fabrication.

Filaments were extruded using the following settings: H4 = 220 °C, H3 = 230 °C, H2 = 235 °C, H1 = 240 °C, corresponding to the progression from the feeding zone (H4) to the nozzle (H1) as shown in Figure 1. The screw speed (3–5 rpm) controlled material throughput, while the fan speed (50%) regulated cooling of the extrudate. To improve filler dispersion, first pass filaments were shredded and re-extruded. Only the filament within 1.75 ± 0.05 mm was collected for subsequent 3D printing.

### 2.3. 3D Printing of Samples

**Figure 2:**
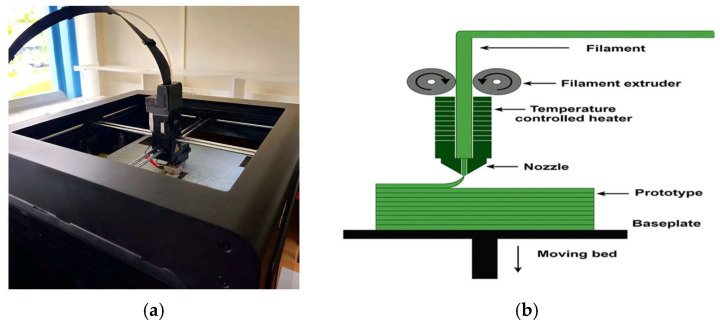
(**a**) Zortrax M300 3D printer used to fabricate ABS and composite samples. (**b**) Schematic representation of the FDM printing process.

Samples were printed using a Zortrax M300 3D printer (Zortrax S.A., Olsztyn, Po-land) equipped with a 0.4 mm nozzle and operated in a fully enclosed chamber (Figure 2a). The printer uses the fused deposition modeling (FDM) technique, in which softened filament is extruded through a heated nozzle and deposited layer-by-layer to form the final structure (Figure 2b).

A customized ABS printing profile was used for all materials. The nozzle temperature was set to 280 °C and the baseplate temperature to 90 °C, with a print speed of 30 mm/s and 5% cooling fan, a 0.29 mm layer height, and 100% infill to ensure consistent bulk density of attenuation measurements. A raft was used for every print to improve first layer adhesion, particularly for composites with increased viscosity.

**Figure 3:**
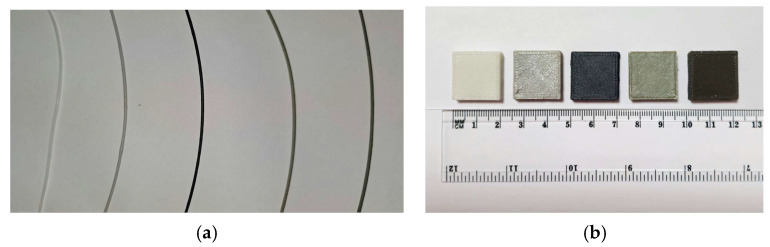
(**a**) Extruded ABS-based composite filaments; (**b**) 3D-printed samples (20 mm × 20 mm × 5 mm) of each material formulation. Materials are arranged from left to right as follows: ABS, ABS–5BN, ABS–30W, ABS–10Gd_2_O_3_, and ABS–30Bi_2_O_3_.

All composite formulations were successfully extruded into 1.75 mm filaments suit-able for FDM printing (Figure 3). However, it should be noted that the incorporation of functional fillers (BN, W, Gd_2_O_3_, and B_i2_O_3_) into the ABS matrix presented several chal-lenges during filament extrusion and 3D printing, primarily related to flow characteristics and print density, consistent with difficulties reported in the literature for high filler con-tent composites [50,53,56].

The physical density of all printed samples was measured to verify uniform bulk properties prior to attenuation testing using the Archimedes method. Each sample was weighed in air and then submerged in distilled water. The values were then compared with theoretical densities to evaluate printing accuracy and material distribution consistency.

$$\rho =\left(\frac{m_{s}}{m_{s}-m_{w}}\right)\times \rho_{w}$$

$m_{s}$—The sample’s mass in air;

$m_{w}$—The sample’s mass in distilled water;

$\rho_{w}$ = 1.0 g/cm^3—^ the density of the distilled water.

### 2.4. Mechanical Tests

**Figure 4:**
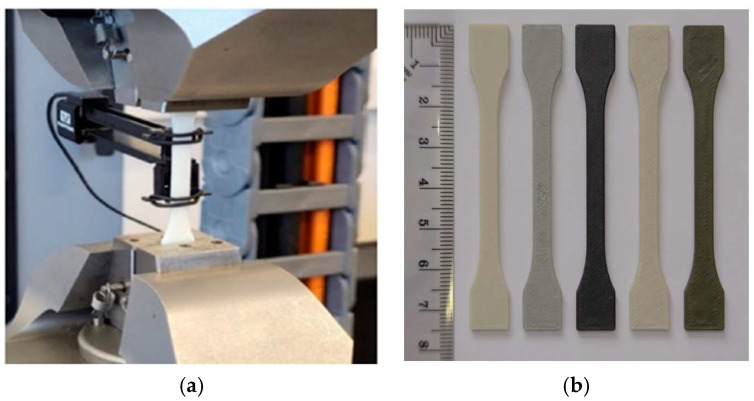
(**a**) Equipment used to perform tensile tests of 3D-printed samples; (**b**) 3D-printed test specimens fabricated from ABS and ABS-based composite filaments used for mechanical characterization. Materials are arranged from left to right as follows: ABS, ABS–5BN, ABS–30W, ABS–10Gd_2_O_3_, and ABS–30Bi_2_O_3_.

Dog-bone-shaped tensile specimens were 3D printed in accordance with ISO 527-2 [60] for the determination of the tensile behavior of plastics (Figure 4b). Mechanical testing was performed using a an ElectroPuls^®^ E10000 Linear-Torsion machine (Instron, MA, USA) according to ISO 527-1:2019 [61] (Figure 4a) at a constant crosshead speed of 1 mm/min. Mechanical response was evaluated based on the recorded stress–strain curves.

### 2.5. Evaluation of Surface Morphology and Topography

Surface topography of some experimental samples has been performed using atomic force microscope (NanoWizard III AFM, JPK Instruments, Bruker Nano GmbH, Berlin, Germany). AFM images were collected using a V-shaped silicon cantilever (spring constant of 3 N/m, pyramidal tip shape, tip curvature radius (ROC) of 10.0 nm, and cone angle of 20°) operating in contact quantitative imaging mode. Data were analyzed using JPKSPM data processing software (Version spm-4.3.25, JPK Instruments, Bruker Nano GmbH, Berlin, Germany).

Surface and cross-sectional morphology of experimental samples have been assessed using a scanning electron microscope (SEM, Hitachi S-3400 N, Tokyo, Japan) equipped with a secondary electron detector. In addition, elemental composition and elemental mapping of the 3D-printed composite polymers, reinforced with metal oxide nanoparticles, were investigated with energy-dispersive X-ray spectroscopy (EDX, Bruker Quad 5040, Billerica, MA, USA).

To determine the attenuation properties of the in-house produced materials, and to assess the homogeneity of samples and radiation density of fabricated materials, CT scans of experimental samples printed with 100% infill density were analyzed using the software package ImageJ 1.54g (National Institutes of Health, Bethesda, MD, USA). Sample images were acquired by CT (Siemens Light Speed RT16, Erlangen, Germany) using the same exposure parameters (head protocol; tube voltage 120 kV; current 335 mA).

### 2.6. Radiation Attenuation Measurements

**Figure 5:**
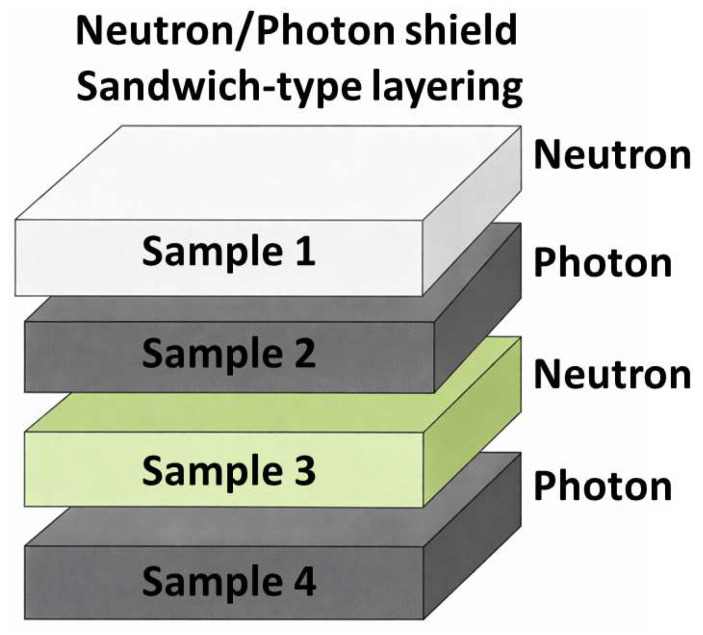
Multilayer configuration used in attenuation experiments, illustrating the sequential arrangement of composite samples for mixed-field photon–neutron shielding evaluation.

Radiation attenuation was evaluated for all samples using two irradiation conditions: X-rays (120 kVp) and mixed neutron–photon fields from a Pu–Be source. For both modalities, a reference dose was measured in the direct beam, followed by the transmitted dose with the sample in place. All exposures delivered a nominal 1 Gy. Measurements were performed for individual samples and for a multilayer stacked configuration (Figure 5), where the incident beam entered the materials in the following order:

1. ABS–5BN;
2. ABS–30W;
3. ABS–10Gd_2_O_3_;
4. ABS–30Bi_2_O_3_.

**Figure 6:**
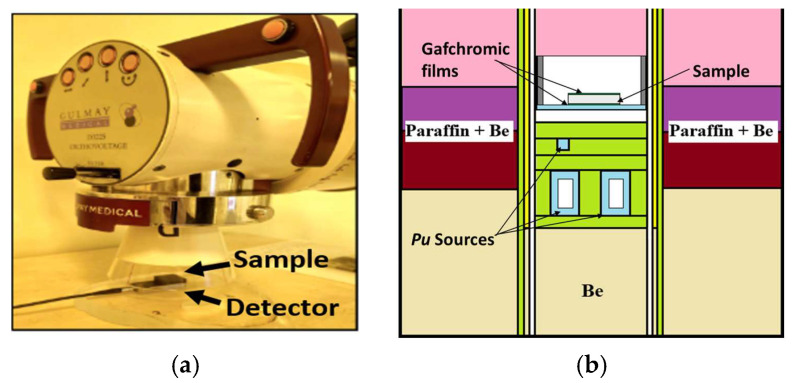
(**a**) Experimental setup for 120 kVp X-ray attenuation measurements. (**b**) Schematic of the Pu–Be mixed neutron–photon irradiation geometry.

Low-energy photon attenuation was examined using a Gulmay D3225 X-ray unit (Gulmay Medical Ltd., Surrey, UK) operated at 120 kVp. Dose readings were obtained with a Piranha dosimetry system (RTI Group, Mölndal, Sweden) (Figure 6a). Gafchromic EBT3 films (Ashland, Wilmington, DE, USA), calibrated for this beam quality, were placed at the same measurement position to provide independent verification of detector readings.

Mixed-field attenuation was assessed using a Pu–Be source (4.5 × 10^7^ n/s) with a central channel neutron flux of 7.12 × 10^4^ n/cm^2^/s, consisting of 68% thermal, 9% epithermal, and 23% fast neutrons. Attenuation was quantified using EBT3 films calibrated for current beam and placed directly behind each sample along the central irradiation axis (Figure 6b).

### 2.7. Data Analysis and Attenuation Calculations

Because the detectors used in this study (Piranha system and EBT3 films) measure absorbed dose, attenuation was evaluated directly from the ratio of transmitted to reference dose. Under narrow-beam conditions, this dose ratio is proportional to the primary beam intensity, allowing the Beer–Lambert law to be applied without additional scatter corrections. The attenuation percentage both for X-ray and Pu-Be source irradiation was calculated as

$$\%A=\left(1-\frac{D_{t}}{D_{0}}\right)\times 100$$

where *D*_0_ is the reference dose measured directly and *D_t_* is the transmitted dose measured with the sample in place.

To allow comparison between shielding configurations with different total thicknesses, a thickness-normalized attenuation parameter, *k_eff_*, was additionally calculated based on the measured dose ratio. This parameter was defined as

$$k_{eff}=-\frac{ln\left(\frac{D_{t}}{D_{0}}\right)}{d_{tot}}$$

where *d_tot_* is the total thickness of the tested shielding configuration expressed in centimeters; for multilayer stacks, *k_eff_* represents an empirical, order-dependent metric that reflects the overall attenuation behavior of the specific layer sequence under the given irradiation conditions.

For photon attenuation measurements, the experimental mass attenuation coefficient (MAC) of each composite was obtained using

$${\left(\frac{\mu }{\rho }\right)}_{comp}=-\frac{1}{\rho_{comp}x}ln\left(\frac{D_{t}}{D_{0}}\right)$$

To compare the photon attenuation performance of the composites with lead, the relative attenuation efficiency (*RAE*) was calculated using

$$RAE\left(\%\right)=\left(\frac{{\left(\frac{\mu }{\rho }\right)}_{comp}}{{\left(\frac{\mu }{\rho }\right)}_{Pb}}\right)\times 100$$

This parameter expresses the photon shielding capability of each material as a percentage of lead’s attenuation efficiency at the same energy.

Theoretical attenuation for photons was estimated using NIST XCOM MAC values at 50–60 keV, corresponding to the effective energy of the 120 kVp polyenergetic X-ray beam. Using the monoenergetic value at 120 keV would underestimate attenuation because it does not represent the actual beam spectrum.

## 3. Results and Discussion

### 3.1. Extrusion of Filaments and Their Characterization

As shown in Table 2, all composite filaments exhibited reduced surface quality (grainy or slightly textured) and some flow issues (pulsing or minor clogging) compared to pure ABS, which had a smooth surface and flow. This degraded flowability in filled polymers is expected, as incorporating shielding fillers typically leads to high melt viscosity, hindering the extrusion process [57,59].

Specifically, the W and Bi_2_O_3_ composites suffered from a pervasive “pulsing flow”, suggesting viscosity issues that were mitigated during printing by increasing the extrusion temperature from 265 °C to 280 °C to achieve a more stable melt flow (Table 3). This adjustment aligns with the principle that higher extrusion temperatures are often necessary to maintain flow quality when printing composites.

Optimization of printing parameters was essential to counter the inherent material challenges and achieve functional density, confirming that precise parameter adjustment is critical for high quality composite printing, as noticed in previous works as well [50,55,56,57,58,59]. The adjustments detailed in Table 3, such as reducing print speed and controlling the extruder flow rate, addressed issues like surface roughness and over-extrusion, factors known to negatively impact layer uniformity and lead to defects.

Despite the optimization, all printed composites showed slightly lower measured densities than theoretical values (Table 4), a typical outcome of FDM processes where micro-voids and incomplete interlayer fusion reduce bulk compactness. The highest discrepancy occurred in the W and Bi_2_O_3_ composites (−5.7% and −5.9%, respectively), materials that also exhibited the most significant extrusion flow issues (Table 2). This connection highlights that flowability issues during printing can result in the formation of pores and voids in the final components as mentioned also by other research groups [50,53,57,58,59].

Similar density deficits (95–99% of theoretical) were reported for W- and B_4_C-filled PEEK composites by Wu et al. [54,57], who attributed the loss to viscosity-driven limitations in melt flow. These results confirm that densification limitations are inherent to highly filled 3D-printed shielding materials and must be considered when interpreting attenuation trends.

### 3.2. Characterization of 3D-Printed Samples

It should be noted that the main focus of this article was the evaluation of the mixed-beam attenuation properties of newly developed and fabricated 3D printing materials. It is assumed that this first step is absolutely necessary for the selection of the best attenuating composites and multilayer compositions for the real fabrication of shielding constructions and for avoiding the unnecessary fabrication and detailed investigation of the morphological, mechanical, and thermal properties of less suitable samples. Examples of the rough investigation of ABS reinforced with Bi_2_O_3_ particles are provided below.

**Figure 7:**
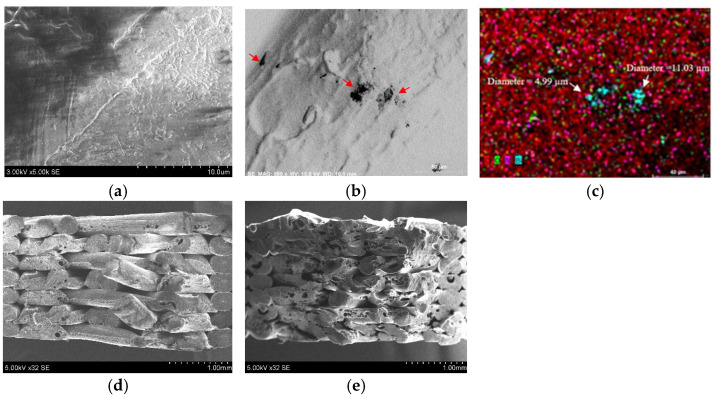
SEM micrographs of ABS surface (**a**) and fracture surface resulting from tensile testing (**d**) and ABS–30Bi_2_O_3_ surface (**b**) with EDX image (**c**) and fracture surface resulting from tensile testing (**e**). Attention: magnification in different images is different.

Scanning electron microscopy (SEM) analysis was carried out on the surfaces and fractured surfaces of the ABS and ABS–30Bi_2_O_3_ composites, to identify mechanisms believed to be responsible for the observed changes in the fracture surface. The corresponding SEM images and EDX map for the ABS–30Bi_2_O_3_ composite are provided in Figure 7.

As can be seen from Figure 7, the added filler content leads to an increase in elastic fracture, with more polymer spikes visible on the fracture surface compared to pure ABS. Moreover, in samples with 30% filler content, filler agglomerations are visible (Figure 7c). These agglomerations appear to influence the brittleness of composites compared to pure ABS. These findings agree with the tensile stress results provided in Section 3.3 of this article.

Additionally, it was observed that for the high filler loadings, as it was in the case of ABS–30Bi_2_O_3_, the potential agglomeration of filler particles likely caused small, air-filled voids and pores to form in the 3D-printed samples, indicating the influence of the extruded polymer composite filament’s inhomogeneity, both in terms of filler distribution within the polymer matrix and the filament’s shape.

**Figure 8:**
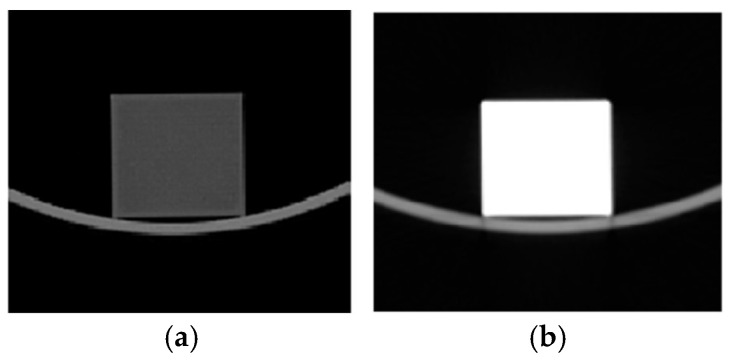
CT scans of experimental samples: (**a**) ABS, (**b**) ABS–30Bi_2_O_3_.

The radiation density of the material plays an important role when analyzing the attenuation properties of the materials. An investigation of the CT scans of the experimental samples measured across five consecutive sample slices was performed (Figure 8) and evaluated using ImageJ program package, which indicated a very high radiation density of 3071 ± 9.584 HU for ABS–30Bi2O3 as compared to 133.069 ± 17.459 for ABS. It should be mentioned that radiation density was calculated specifically for photon beams.

**Figure 9:**
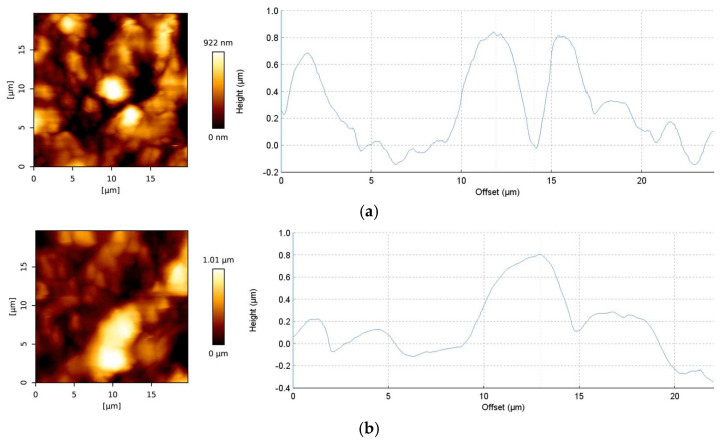
The 2D surface topography and roughness height profiles of ABS (**a**) and ABS-30Bi_2_O_3_ (**b**) samples.

The results of the performed AFM measurements were in line with the discussed SEM results. Increased surface roughness of 3D-printed ABS polymers containing 30% of Bi_2_O_3_ and less uniform surface profile was observed, compared with printed pure ABS samples (Figure 9). Specifically, the average roughness R_a_ (arithmetic average of the absolute values of the profile heights over the evaluation length) value of 177.4 nm and root mean square roughness R_q_ (RMS average of the profile heights over the evaluation length) value of 229.6 nm for ABS–30Bi_2_O_3_ were observed in comparison with 163.0 nm and 209.6 nm for pure ABS.

More detailed analysis of ABS containing different concentrations of Bi_2_O_3_ can be found in our previous paper [58].

### 3.3. Initial Mechanical Testing Results of Experimental Samples

**Figure 10:**
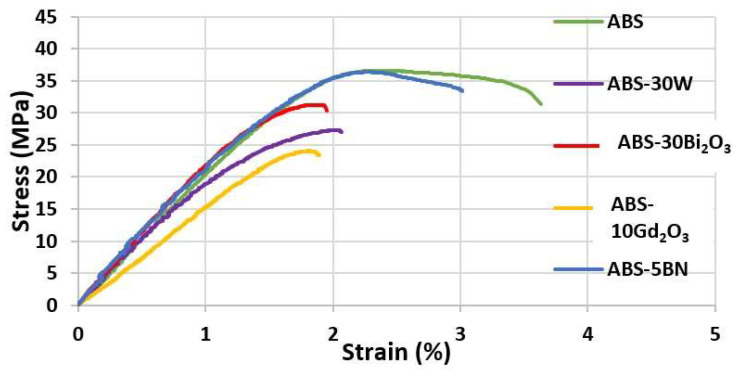
Stress–strain curves of the investigated initial ABS—based composites.

Tensile tests of all experimentally fabricated 3D-printed samples have been investigated and the results are shown in the form of stress–strain curves in Figure 10. It is evident that all additives incorporated into the ABS polymer matrix reduced the plasticity of ABS. The lowest impact was observed for BN filler: the ABS–5BN composite was less brittle, but had almost the same UTS value of 36.35 MPa as ABS. Young’s modulus values for ABS–5BN and ABS–30Bi_2_O_3_ composites were almost the same at 2133 MPa and slightly higher than the value of 2083 MPa for pure ABS. ABS–30Bi_2_O_3_ and ABS–10Gd_2_O_5_ were most brittle from all investigated samples. In addition, the ABS–10Gd_2_O_5_ composite indicated the lowest value of 1552 MPa for Young’s modulus.

The performed investigation revealed that the mechanical properties (tensile) of ABS are reduced after the incorporation of fillers into the matrix. Experimental composites from the investigated set can be compatible when adjusting their mechanical properties for the fabrication of layered shielding constructions accordingly.

### 3.4. Attenuation Properties of the Experimental 3D-Printed Samples

**Figure 11:**
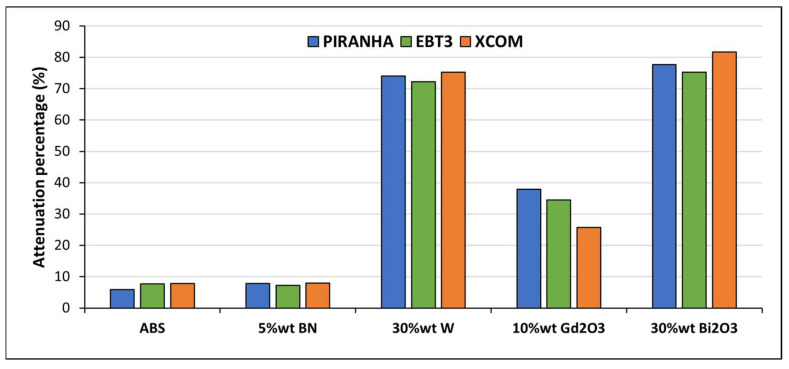
Attenuation performance of ABS-based composite samples at 120 kVp measured using Piranha, EBT3 film, and XCOM calculations.

The attenuation of X-rays in the energy range of 120 kVp is governed primarily by the photoelectric effect, which scales steeply with the atomic number (Z^4 − 5^) of the absorbing material. This fundamental principle is directly reflected in the measured attenuation percentage of the shielding composite materials (Figure 11). The material with the lowest measured performance, ABS–5BN, showed only ~7.98% attenuation. This result is expected because boron compounds (such as boron nitride) consist of low-Z elements and thus offer minimal photon shielding. A substantial increase in shielding was observed with high-Z fillers. The ABS–30W composite achieved ~72–75% attenuation across Piranha, EBT3, and XCOM, reflecting tungsten’s high photoelectric interaction probability at energies around 120 kVp. The ABS–30Bi_2_O_3_ composite demonstrated the highest overall attenuation (~78–82%), which is consistent with bismuth’s higher atomic number and its K-edge proximity (90.5 keV) to the peak of the 120 kVp X-ray spectrum, enhancing photoelectric absorption [31,54]. The ABS–10Gd_2_O_3_ composite provided ~35–40% attenuation which is lower than W and Bi_2_O_3_ due to both its lower weight fraction and lower Z.

Piranha-based measurements were selected as the primary attenuation quantification method, with EBT3 film used for independent validation; the close agreement between the two confirms the stable fabrication and acceptable sample uniformity. XCOM calculations showed some discrepancies compared to measurement results, which are expected because XCOM assumes an ideal, homogeneous material and a strictly monoenergetic beam, while our measurements were conducted in a polychromatic spectrum and are influenced by FDM-related microstructural variations.

The measured mass attenuation coefficients (MACs) further confirmed the strong dependence of the shielding performance on the filler atomic number and loading (Table 5) [39,59]. Pure ABS demonstrated the lowest MAC (0.12 cm^2^/g), reflecting the limited photon interaction probability of low-Z polymers [51]. The slight increase observed for ABS–5BN (0.16 cm^2^/g) is consistent with BN’s modest contribution to photoelectric absorption at diagnostic energies. In contrast, the incorporation of high-Z fillers produced substantial increases in the MACs.

ABS–30W exhibited a MAC of 1.95 cm^2^/g, almost an order of magnitude higher than pure ABS, aligning with the literature reporting exponential MAC growth with increasing W content in polymer matrices. Studies involving W-based polymer composites consistently demonstrate improved radiation shielding performance and are recognized for manufacturing complex 3D-printed parts and wearable equipment where toughness and low toxicity are required. The highest MAC was obtained for ABS–30Bi_2_O_3_ (2.30 cm^2^/g), consistent with the superior attenuation of bismuth-containing composites observed in previous studies (Pavlenko et al., 2019 [36]; Shabib et al., 2025 [38]), as bismuth is favored as a lead-free replacement owing to its high atomic number (Z = 83) and non-toxic nature, offering comparable shielding efficiency. ABS–10Gd_2_O_3_ (0.88 cm^2^/g) showed intermediate MAC values, reflecting both the lower filler loading and Gd’s reduced photoelectric contribution compared to W and Bi [51].

**Figure 12:**
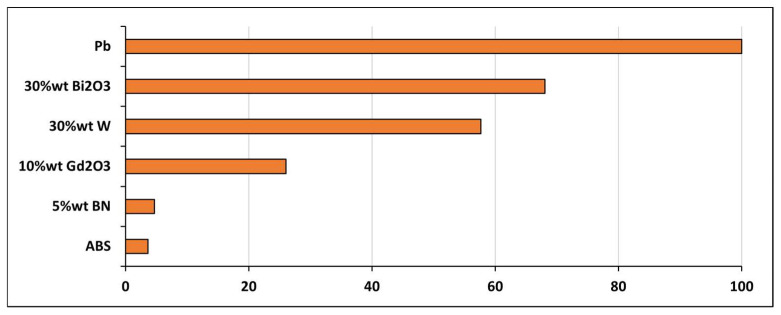
Relative attenuation efficiency (RAE) of ABS-based composites at 120 kVp, benchmarked against lead.

The RAE results were found to clearly distinguish the shielding capabilities of the composites (Figure 12). Lead was measured as the reference material (100%), while ABS–30Bi_2_O_3_ was determined to reach ~68%, representing the highest efficiency among the lead-free samples. ABS–30W was observed at ~58%, and ABS–10Gd_2_O_3_ at ~26%. Very low efficiencies (<5%) were recorded for ABS–5BN and pure ABS. Generally, the RAE values were shown to align with the attenuation and MAC trends.

**Figure 13:**
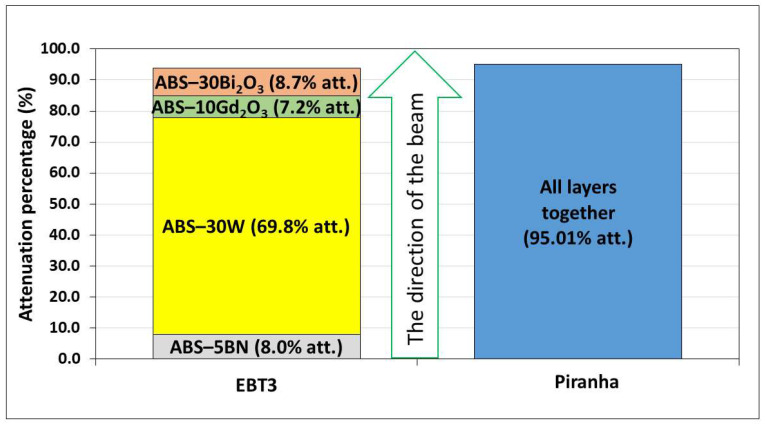
Individual contribution of each layer in the multilayer composite system to total attenuation at 120 kVp (EBT3), compared with overall attenuation measured by Piranha.

The multilayer configuration was found to produce a cumulative attenuation of ~95%, as shown in Figure 13, demonstrating a strong synergistic effect among the individual layers. Although Bi_2_O_3_ showed the highest attenuation when measured as a single material, the W-containing layer was observed to contribute the largest share within the multilayer system.

This can be explained by the fact that attenuation in a stacked configuration is not determined solely by the intrinsic attenuation coefficient, but by how much additional attenuation each layer provides after the preceding layers have already removed part of the spectrum. Since W interacts strongly with both low- and mid-energy photons, it removes a substantial portion of the incident beam before significant spectral hardening occurs. Consequently, the remaining layers encounter a reduced and hardened spectrum, causing their incremental contributions, particularly that of Bi_2_O_3_, to appear smaller despite their high individual performance.

The thickness-normalized attenuation values in Table 6 further illustrate that the multilayer configuration is not optimized to maximize photon attenuation per unit thickness, but rather to combine materials with distinct interaction characteristics within a single structure. Because the stack intentionally includes both high-Z photon-attenuating layers and lower-Z functional layers, the resulting *k_eff_* represents an averaged response over materials with different attenuation efficiencies. Consequently, a lower *k_eff_* for the multilayer is expected and does not contradict its superior cumulative attenuation, but instead reflects the trade-off between per-thickness efficiency and multifunctional shielding design.

**Figure 14:**
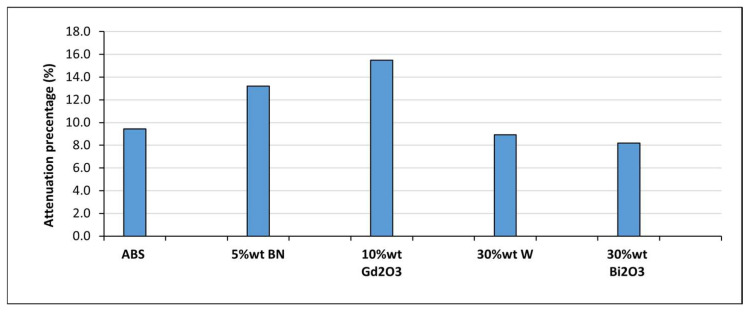
Mixed-field attenuation of ABS-based composites measured with EBT3 film under Pu–Be source irradiation.

The mixed-field attenuation under Pu-Be source irradiation for single sample results demonstrated behavior distinct from the X-ray measurements, reflecting the fundamentally different interaction mechanisms governing neutrons and secondary photons. In general, attenuation remained modest (8–16%), as can be observed in Figure 14, which is expected for thin polymer-based samples exposed to a broad neutron spectrum. The highest reduction was observed for ABS–10Gd_2_O_3_, consistent with gadolinium’s exceptionally high thermal neutron capture cross-section [44]. A similar, though smaller, enhancement was recorded for ABS–5BN, consistent with boron’s capacity to absorb thermalized neutrons [42]. In contrast, ABS–30W and ABS–30Bi_2_O_3_ exhibited only limited attenuation, aligning with the fact that high-Z fillers contribute minimally to fast-neutron moderation or capture. However, some measurable attenuation was still detected in these composites because the EBT3 film recorded an integrated response to both neutron-induced dose and accompanying secondary photon dose. As a result, photon interactions within W- and Bi-containing samples contributed to the measured film response, and the reported attenuation values therefore represent an effective mixed-field dose reduction rather than a quantitative separation of neutron capture and photon attenuation mechanisms.

Furthermore, it should be noted that ABS, while offering excellent printability and structural stability, contains a lower hydrogen density compared to polyethylene-based matrices, which are more effective for fast neutron moderation. Quantitatively, ABS contains approximately 7–8 wt% hydrogen, compared to ~14.4 wt% for high-density polyethylene, corresponding to roughly two times lower hydrogen atom density [49,59]. As a result, neutron slowing in the present ABS-based samples was inherently limited, particularly given the relatively small sample thickness of 5 mm, which provided an insufficient path length for extensive moderation prior to thermal neutron capture. Consequently, the observed mixed-field attenuation was dominated by thermal neutron capture rather than moderation effects. Future work will therefore explore hydrogen-rich matrices such as HDPE in combination with neutron-absorbing fillers to enhance fast neutron moderation while retaining the advantages of additive manufacturing.

The multilayer configuration was designed based on established neutron–photon shielding principles, where neutron-absorbing and photon-attenuating materials are arranged in an alternating sequence. Neutron capture reactions in boron- and gadolinium-containing layers are accompanied by the emission of secondary gamma radiation, causing these layers to act as secondary photon sources. The inclusion of adjacent high-Z layers therefore enables the attenuation of both primary and secondary photons. Repeating neutron–photon stages allow the progressive reduction in residual radiation components, providing more efficient mixed-field attenuation within a compact structure [30,41].

**Figure 15:**
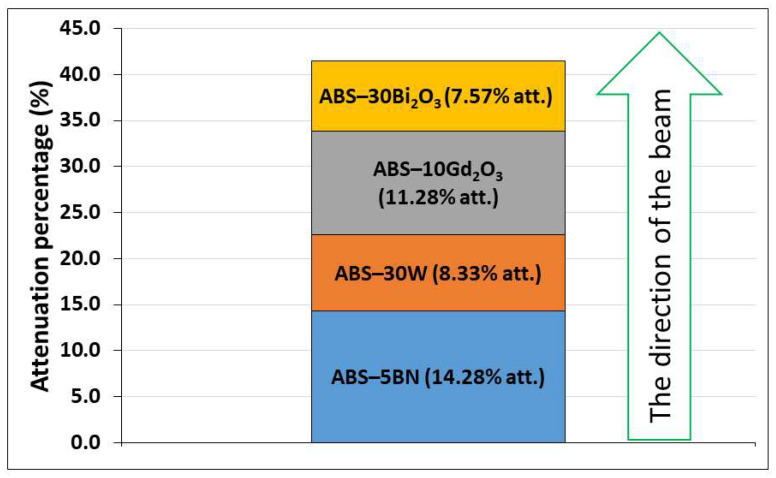
Individual contribution of each layer in the multilayer composite system to total attenuation of Pu–Be source mixed-field irradiation, based on EBT3 film response.

Under Pu–Be irradiation, the multilayer exhibited a cumulative attenuation composed of distinct contributions from each layer (Figure 15), reflecting the differing neutron interaction mechanisms of the fillers [57]. The BN-containing layer exhibited the largest effective attenuation (~14.3%), which is consistent with the strong affinity of boron for absorbing thermalized neutrons. The ABS–10Gd_2_O_3_ layer followed (~11.3%), supported by gadolinium’s exceptionally high thermal neutron capture cross-section. These two layers dominated the overall response, suggesting that neutron capture-related processes played a dominant role in the effective mixed-field attenuation. The noticeable, though smaller, contributions from the ABS–30W and ABS–30Bi_2_O_3_ layers (~8.3% and ~7.6%, respectively) show that photon-sensitive materials still played a role in the mixed field, consistent with the fact that EBT3 integrates both a neutron- and photon-induced dose. Because the layers were arranged in an alternating neutron–photon–neutron–photon attenuating materials sequence, the observed pattern reflects the interplay between moderation/capture processes and photon attenuation rather than a simple upstream–downstream depletion effect. The results therefore highlight that, in this geometry, neutron-active fillers largely govern dose reduction, while high-Z layers mainly provide an additional, secondary contribution linked to the photon component of the Pu–Be field.

The thickness-normalized attenuation values in Table 7 indicate that neutron-active single layers (ABS–5BN and ABS–10Gd_2_O_3_) exhibit higher *k_eff_* than photon-dominant composites, reflecting the dominance of thermal neutron capture in the mixed field. The multilayer configuration shows a lower *k_eff_* despite higher cumulative attenuation, as its thickness is distributed among materials with different neutron and photon interaction roles. Moreover, neutron capture in BN- and Gd-containing layers is accompanied by secondary gamma emission, which contributes to the dose recorded by EBT3 and limits the apparent per-thickness attenuation.

Future research may focus on optimizing layer sequencing and thickness ratios to enhance the mixed-field attenuation efficiency while minimizing material usage. Incorporating higher loadings of neutron-active fillers or exploring alternative isotopes with strong capture cross-sections could further improve performance. Detailed Monte Carlo modeling should be employed to resolve the individual neutron and photon contributions measured by EBT3 films. Additionally, evaluating the mechanical integrity, thermal behavior, and long-term stability under prolonged irradiation would support the development of application-ready, lead-free multilayer shields.

## 4. Conclusions

This work demonstrated that ABS-based composites containing BN, W, Gd_2_O_3_, and Bi_2_O_3_ can be successfully processed into filaments and 3D-printed into functional shielding structures, confirming the feasibility of additive manufacturing for lead-free radiation protection applications. Although high filler loadings introduced viscosity-related challenges and minor porosity, careful adjustment of the extrusion and printing parameters enabled the fabrication of dense, mechanically stable components. Under 120 kVp X-ray irradiation, W- and Bi_2_O_3_-filled composites showed high attenuation efficiency for the investigated formulations, and the multilayer architecture achieved ~95% attenuation, illustrating the capability of 3D printing to tailor material distribution within complex shielding designs. Mixed-field Pu–Be measurements highlighted the complementary behavior of neutron-active and photon-active fillers, demonstrating that customized layer sequencing can be strategically used to target specific radiation environments. In conclusion, these results show that 3D printing enables lightweight, geometry-flexible, and compositionally tunable shielding materials, offering a promising pathway toward application-ready alternatives to conventional lead-based systems.

## Figures and Tables

**Table 1:** Composite formulations and their physical parameters.

Material	Composition (wt%)	Dominant Element (Z)	Density of Functional Filler (g/cm^3^)
ABS–5BN	95% ABS/5% BN	B (Z = 5)	2.1
ABS–30W	70% ABS/30% W	W (Z = 74)	19.3
ABS–10Gd_2_O_3_	90% ABS/10% Gd_2_O_3_	Gd (Z = 64)	7.41
ABS–30Bi_2_O_3_	70% ABS/30% Bi_2_O_3_	Bi (Z = 83)	8.9

**Table 2:** Filament extrusion characteristics for ABS-based composites.

Material	Filament Diameter (mm)	Surface Quality	Issues Observed
ABS	1.75 ± 0.03	Smooth	No issues, smooth flow
ABS–5BN	1.75 ± 0.05	Grainy	Minor clogging, pulsing flow
ABS–30W	1.75 ± 0.05	Slightly textured	Pulsing flow
ABS–10Gd_2_O_3_	1.75 ± 0.04	Grainy	Minor clogging, occasional filament breakage
ABS–30Bi_2_O_3_	1.75 ± 0.05	Slightly textured	Minor clogging, pulsing flow

**Table 3:** Printing issues encountered and corresponding parameter adjustments.

Issue Observed	Material(s) Affected	Adjustment Made	Result
Slight warping at edges	ABS	Bed temperature increased from 80 °C to 90 °C	Warping eliminated
Unstable or pulsing extrusion flow	W and Bi_2_O_3_ containing composites	Extrusion increased from 265 °C to 280 °C	More stable melt flow
Over-extrusion and pressure buildup	W and Bi_2_O_3_ containing composites	Extruder flow rate set to 45%	Smoother, more controlled deposition
Surface roughness at 50 mm/s	All composite samples	Print speed reduced to 30 mm/s	Improved layer uniformity
Weak first-layer adhesion	BN and Gd_2_O_3_ containing composites	Cooling fan reduced to 5%	Better first-layer bonding

**Table 4:** Theoretical and measured densities of printed materials.

Material	Theoretical Density (g/cm^3^)	Measured Density (g/cm^3^)	Difference (%)
ABS	1.050	1.022	−2.7
ABS–5BN	1.077	1.033	−4.3
ABS–30W	1.466	1.387	−5.7
ABS–10Gd_2_O_3_	1.149	1.108	−3.7
ABS–30Bi_2_O_3_	1.428	1.349	−5.9

**Table 5:** Mass attenuation coefficients (MACs) of ABS-based composites at 120 kVp.

Material	MAC (cm^2^/g)
ABS	0.12
ABS–5BN	0.16
ABS–30W	1.95
ABS–10Gd_2_O_3_	0.88
ABS–30Bi_2_O_3_	2.30

**Table 6:** Thickness-normalized attenuation parameter (*k_eff_*) for single-layer composites and the multilayer configuration under 120 kVp X-ray irradiation.

Material	Total Thickness (cm)	*k_eff_* (cm^−1^)
ABS	0.5	0.123
ABS–5BN	0.5	0.164
ABS–30W	0.5	2.694
ABS–10Gd_2_O_3_	0.5	0.951
ABS–30Bi_2_O_3_	0.5	2.997
Multilayer (BN/W/Gd_2_O_3_/Bi_2_O_3_)	2.0	1.499

**Table 7:** Thickness-normalized attenuation parameter (*k_eff_*) for single-layer composites and the multilayer configuration under Pu–Be mixed-field irradiation.

Material	Total Thickness (cm)	*k_eff_* (cm^−1^)
ABS	0.5	0.189
ABS–5BN	0.5	0.279
ABS–30W	0.5	0.189
ABS–10Gd_2_O_3_	0.5	0.325
ABS–30Bi_2_O_3_	0.5	0.167
Multilayer (BN/W/Gd_2_O_3_/Bi_2_O_3_)	2.0	0.264

## Data Availability

The original contributions presented in this study are included in the article. Further inquiries can be directed to the corresponding authors.
